# The Evolution of Invasiveness in Garden Ants

**DOI:** 10.1371/journal.pone.0003838

**Published:** 2008-12-03

**Authors:** Sylvia Cremer, Line V. Ugelvig, Falko P. Drijfhout, Birgit C. Schlick-Steiner, Florian M. Steiner, Bernhard Seifert, David P. Hughes, Andreas Schulz, Klaus S. Petersen, Heino Konrad, Christian Stauffer, Kadri Kiran, Xavier Espadaler, Patrizia d'Ettorre, Nihat Aktaç, Jørgen Eilenberg, Graeme R. Jones, David R. Nash, Jes S. Pedersen, Jacobus J. Boomsma

**Affiliations:** 1 Centre for Social Evolution, Department of Biology, University of Copenhagen, Copenhagen, Denmark; 2 Evolution, Behaviour & Genetics, Biology I, University of Regensburg, Regensburg, Germany; 3 Chemical Ecology Group, School of Physical and Geographical Sciences, Keele University, Keele, United Kingdom; 4 Institute of Ecology, Innsbruck University, Innsbruck, Austria; 5 Department of Integrative Biology and Biodiversity Research, Boku, University of Natural Resources and Applied Life Sciences Vienna, Vienna, Austria; 6 Department of Forest and Soil Sciences, Boku, University of Natural Resources and Applied Life Sciences Vienna, Vienna, Austria; 7 School of Marine and Tropical Biology, James Cook University, Townsville, Australia; 8 Natural History Museum Görlitz, Görlitz, Germany; 9 Department of Biology, University of Oulu, Oulu, Finland; 10 Dormagen, Germany; 11 Department of Genetics, Federal Research and Training Centre for Forests, Natural Hazards and Landscape, Vienna, Austria; 12 Department of Biology, Trakya University, Edirne, Turkey; 13 Department of Animal Biology, Plant Biology and Ecology, Autonomous University of Barcelona, Bellaterra, Spain; 14 Department of Ecology, University of Copenhagen, Frederiksberg, Denmark; Lund University, Sweden

## Abstract

It is unclear why some species become successful invaders whilst others fail, and whether invasive success depends on pre-adaptations already present in the native range or on characters evolving *de-novo* after introduction. Ants are among the worst invasive pests, with *Lasius neglectus* and its rapid spread through Europe and Asia as the most recent example of a pest ant that may become a global problem. Here, we present the first integrated study on behavior, morphology, population genetics, chemical recognition and parasite load of *L. neglectus* and its non-invasive sister species *L. turcicus*. We find that *L. neglectus* expresses the same supercolonial syndrome as other invasive ants, a social system that is characterized by mating without dispersal and large networks of cooperating nests rather than smaller mutually hostile colonies. We conclude that the invasive success of *L. neglectus* relies on a combination of parasite-release following introduction and pre-adaptations in mating system, body-size, queen number and recognition efficiency that evolved long before introduction. Our results challenge the notion that supercolonial organization is an inevitable consequence of low genetic variation for chemical recognition cues in small invasive founder populations. We infer that low variation and limited volatility in cuticular hydrocarbon profiles already existed in the native range in combination with low dispersal and a highly viscous population structure. Human transport to relatively disturbed urban areas thus became the decisive factor to induce parasite release, a well established general promoter of invasiveness in non-social animals and plants, but understudied in invasive social insects.

## Introduction

Invasive species use man-made transport networks for their global dispersal and often damage native ecosystems by their high rates of population growth after introduction [1]. Despite the ever increasing number of exotic invasives and the massive problems that they cause, the origins of the traits that determine invasive success remain largely unknown [2], [3], because invasive species typically only reveal their destructive potential following a long, inconspicuous lag phase [4]. As a result, fundamental questions in invasive biology are still largely unanswered: Are key invasive traits already present in the native range, and if so, are they only selected in novel ecological environments? Do invasive traits arise as mutations in small founder populations? Or do they originate later when invasive founder populations grow and adapt to their novel habitats?

Invasive ants are among the world's most damaging pests [5] and are now known from more than 10 genera belonging to three subfamilies [6]–[9]. All invasive ants that have been studied express the same ‘invasive ant syndrome’: queens mate in the nest and disperse on foot accompanied by workers (colony budding) rather than alone during mating flights, and colonies occupy multiple interconnected nests with many queens and do not show territorial aggression [6]–[9]. This leads to the emergence of massive supercolonies that may extend over hundreds or even thousands of kilometers [7], [10] and often exterminate native ants, while significantly affecting native invertebrate biodiversity and human economic interests [6], [7]. Some components of this invasive ant syndrome may also be expressed in the >12,000 species of non-invasive ants [11]–[14], or in native populations of invasive ants [15]–[18]. However, these social structures are best described as ‘extended families’ [19] that do not reach the enormous scale and ecological dominance of invasive supercolonies [15], [19]. Despite their short-term ecological success, supercolonial social systems are considered to be evolutionary dead ends [20]–[22], as clades exclusively comprising supercolonial ants are unknown. Whilst their demise over evolutionary time is predictable, the parallel evolutionary origins of supercolonial invasive ant syndromes are poorly understood [23], [24].

**Figure 1:**
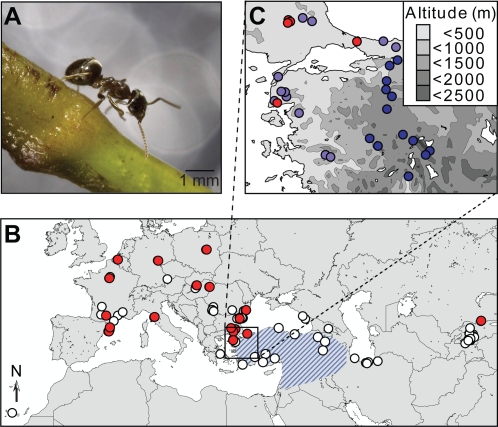
Currently known worldwide distribution of *Lasius neglectus* and *L. turcicus*. (A) *L. neglectus* worker. (B) The study populations (red dots) and all other presently known populations (open dots) of the invasive garden ant *Lasius neglectus* and the partly sympatric distribution of the non-invasive sister species *L. turcicus* (blue stripes). (C) The study area in Turkey with the sampled populations of *L. neglectus* (only occurring in lowland areas; max. 100 m above sea level) and *L. turcicus* in lowland (pale blue dots) and highland (dark blue dots; mean altitude 790 m).

Here, we present a large-scale interdisciplinary study of the invasive ant *Lasius neglectus* (Fig. 1A), which was described as a new species in 1990, when it was found occupying an entire neighborhood in Budapest, Hungary [25]. The first introduction of *L. neglectus* was thus identified 70–100 years later than those of its infamous ‘big sisters’, the Argentine ant and the red imported fire ant, which were detected in 1891 and 1930, respectively [6], [23]. So far about 100 local infestation sites of the invasive garden ant have been recorded (Fig. 1B), most of which were detected after the year 2000 [26], [27]. Similar to many other invasive species, *L. neglectus* has so far only been found to infest disturbed urban habitats such as parks and gardens, where it eradicates most native ants and other insect populations while damaging trees because of the massive aphid cultures that it maintains [28], [29]. Whereas most other known pest ants require warm temperatures to thrive, *L. neglectus* can survive winters with extended frost periods, so that further dispersal into temperate climatic zones seems unavoidable [30], [31]. Asia Minor has been suggested as the most likely region of origin of *L. neglectus* [30] as it co-occurs here with its non-invasive sister-species *L. turcicus* [[32]; Fig. 1B,C].

After the *Lasius turcicus* sister lineage was confirmed [32], the invasive garden ant provided a unique opportunity for comparative analysis of the invasive and pre-invasive traits that determine gene-flow, social structure, recognition and aggression, vulnerability to disease, and thus, ultimately, reproductive success and invasive potential. To this end, we analyzed the morphology, behavior, population genetics, chemical cues and parasite loads of multiple populations of *L. neglectus* and *L. turcicus* to determine the social structure and invasive potential of both species. Our data suggest that the invasive success of *L. neglectus* is based both on pre-adaptations that evolved in the native range and on parasite release during the introduction.

## Results and Discussion

### Origin and Range of *Lasius neglectus*

**Figure 2:**
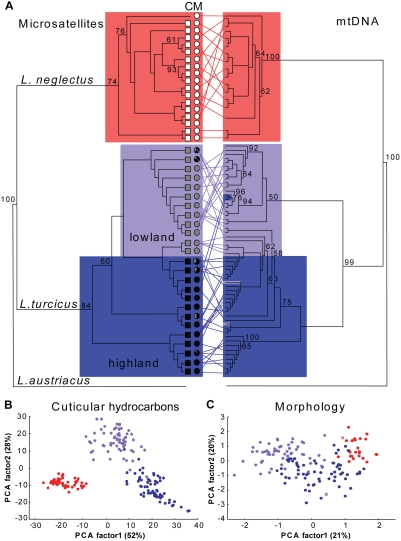
Phylogeography, chemistry and morphology. (A) Microsatellite consensus tree (left: 6 loci, 1362 individuals from 206 nests, 43 populations) and CO1 haplotype neighbor-joining tree (right: 1171 bp, 73 individuals and nests, 43 populations) for *L. neglectus* (red), lowland *L. turcicus* (pale blue) and highland *L. turcicus* (dark blue), with *L. austriacus* as outgroup. Bootstrap values >50% are given except for terminal nodes. Classes of chemical profiles (Fig. 4A) and morphological variation (Methods S5) are indicated as white, grey, and black squares or circles in columns ‘C’ and ‘M’, respectively. (B) Principal Component Analysis of cuticular hydrocarbon profiles of 204 nests (42 populations). (C) Principal Component Analysis of 14 morphological traits of workers from 144 nests (43 populations). The red cross indicates the type specimen of *L. neglectus* and the blue cross that of *L. turcicus*.

We sampled 18 populations assumed to be introduced and invasive across the entire currently known distribution of *L. neglectus*, plus 25 populations of *L. turcicus* in Turkey [supporting information, Fig. S1, Table S1], and found that sympatry is restricted to low altitude habitats (<400 m; Fig. 1C). We constructed phylogenetic trees (Figs. 2A, S2, S3) based on nuclear microsatellite loci and mitochondrial COI haplotypes of the two species with their closest relative, the non-invasive ant *L. austriacus* [32], as outgroup. The trees that we obtained unambiguously support the species status of *L. neglectus* relative to *L. turcicus*, but showed that *L. turcicus* occurs in two distinct clades represented by 12 lowland populations (mean altitude 166 m, max. 370 m a.s.l.) and 13 highland populations (mean 790, max. 1170 m), a result that was also obtained when using the multiple *Lasius* outgroups applied in a recent study by Steiner et al. [32].

The separation of our 43 sampled populations into three biological forms was confirmed by distinct cuticular hydrocarbon profiles [[27]; Figs. 2A,B, Table S2] and worker morphologies (Figs. 2A,C, Table S3). Only the morphology of the highland populations matched the original species description of *L. turcicus* (Fig. 2C; [33]), which implies that the taxonomic status of the lowland form of *L. turcicus* remains to be resolved.

### Reproduction, Social Organization, and Aggression

**Figure 3:**
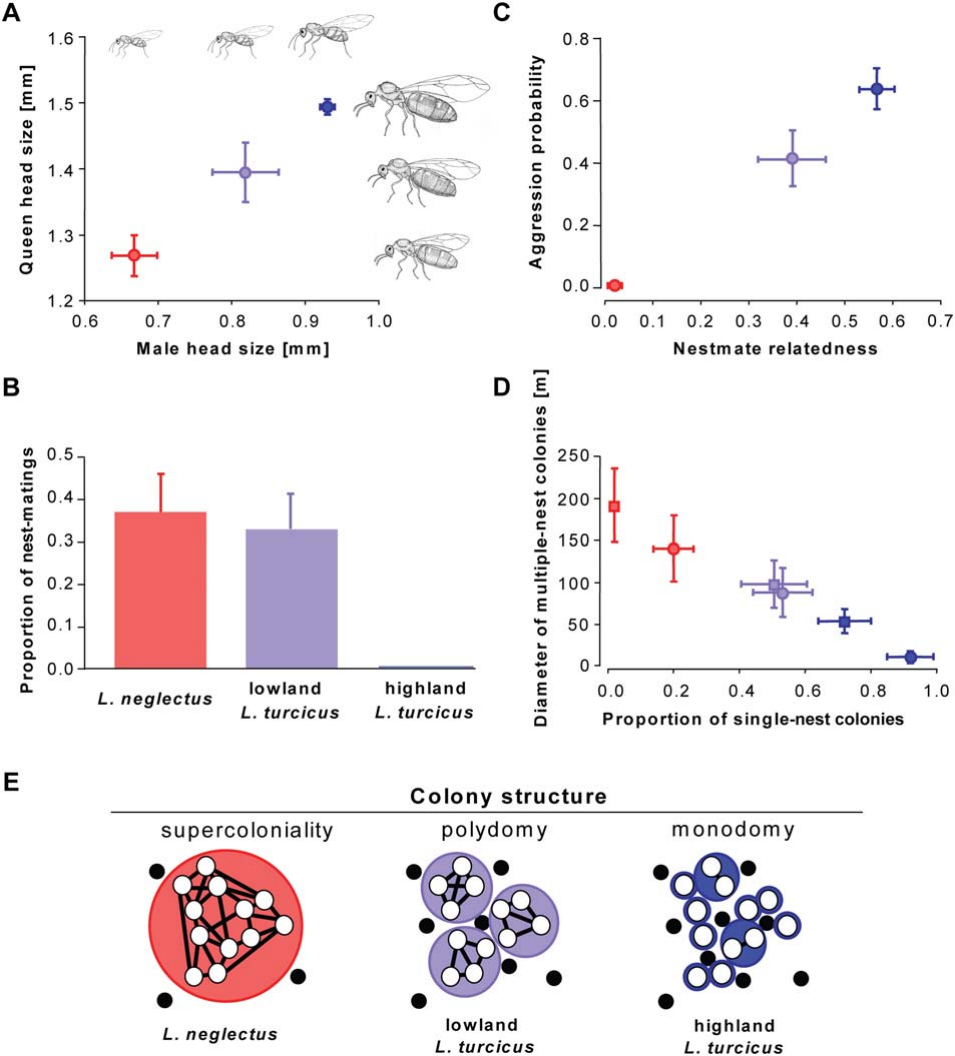
Mating, dispersal, aggression and social organization. (A) Head size of males (*n* = 42) and queens (*n* = 93) of *L. neglectus* (red), lowland *L. turcicus* (pale blue) and highland *L. turcicus* (dark blue). (B) Proportion of successful matings (*n* = 163) in nest-boxes. (C) Aggression probability between nests within populations (528 aggression tests) and nestmate relatedness. (D) Proportion of single-nest (monodomous) colonies, inferred from aggression tests (squares) and Bayesian clustering analysis of microsatellite data (circles) and the maximum diameter of multiple-nest (polydomous) colonies in meters. (E) Schematic diagrams of typical colony organization: neighboring nests (open circles) belong to colonies (colored larger circles) at different scales, allowing different degrees of coexistence with other ant species (black circles) in the matrix. All means are plotted±s.e.m.

Mating within the maternal colony makes invasive ant reproduction independent of the presence of other colonies – a vital advantage when being the first to arrive in a new range. It also means that queens no longer need functional wings and large bodies with substantial fat reserves, as they establish bud nests at walking distance with the support of sibling workers [7]. We indeed found that queens and males of *L. neglectus* are significantly smaller than those of lowland *L. turcicus*, which themselves are smaller than the highland form (Fig. 3A; ANOVA; queens: *F*_2,16_ = 91.5, *P*<0.001; males: *F*_2,16_ = 117.3, *P*<0.001; *P*<0.05 in posthoc Tukey tests of all pairwise comparisons). Male differences appear to concern overall body size only, but queens also differ in body proportions (Fig. 3A): the mesosoma that contains the flight muscles is relatively small in *L. neglectus* and the short wings of this species do not extend beyond the rear end of the body. In contrast, the queens of highland *L. turcicus* have the typical large mesosoma and long wings that characterize *Lasius* species with extensive mating and dispersal flights, whereas queens of lowland *L. turcicus* are intermediate. These differences are thus consistent with exclusive dispersal by colony budding and the loss of airborne dispersal in *L. neglectus* [34], and with lowland *L. turcicus* being a relatively poor flight disperser relative to highland *L. turcicus*.

Mating experiments performed with freshly collected sexuals in the field corroborated our morphological findings. Neither queens nor males of highland *L. turcicus* made any attempts to mate in 106 nest-box trials, but matings were frequently observed in both *L. neglectus* (10/27 trials) and lowland *L. turcicus* (11/33 trials; Fig. 3B). Additional trials in which queens and males of different forms were mixed, showed that highland *L. turcicus* queens mated with males of both *L. neglectus* (*n* = 8) and lowland *L. turcicus* (*n* = 29) in an overall proportion (38%) similar to that observed for matings within *L. neglectus* and lowland *L. turcicus* (χ^2^ = 0.5, d.f. = 2, *P* = 0.80). However, combining males of highland *L. turcicus* with queens of *L. neglectus* (*n* = 7) or lowland *L. turcicus* (*n* = 51) never produced any matings, suggesting that the males of highland *L. turcicus* need to fly before being able to copulate. These results mean that invasive pre-adaptations towards mating in the nest are present in highland *L. turcicus* (queens do not need to fly in order to copulate) and, in particular, in lowland *L. turcicus*, where at least some individuals of both sexes are willing and able to mate without dispersing. This suggests that part of the lowland *L. turcicus* populations are propagated by intra-nest mating and colony budding, but without being invasive, a social structure that also occurs in other ant species with multiple queens per nest [20]. Nest excavations confirmed that queen numbers are high in *L. neglectus* (mean 4.3±1.1 s.e.m.) and lowland *L. turcicus* (5.5±1.7), while highland *L. turcicus* is monogynous (1.0±0.0).

Aggression is typically low between nest buds of ant species with multiple queens, and often completely absent over large areas in invasive ants [7]. No aggression among workers of different nests within the same population could be detected in *L. neglectus*, whereas aggression was high in highland *L. turcicus,* and intermediate but significant in lowland *L. turcicus* (Fig. 3C). The absence of inter-nest aggression in *L. neglectus* allows free mixing of individuals among all nests of a local population and implies that nestmates are expected to be as (un)related as randomly sampled individuals in the population. Our regression relatedness estimates confirmed this (*r* = 0.022±0.013 s.e.m.; difference from zero: *t*_15_ = 1.66, *P* = 0.12). In lowland *L. turcicus,* relatedness was moderately high (0.392±0.070), whereas relatedness in highland *L. turcicus* reached levels that are consistent with territorial colonies headed mostly by a single queen (0.561±0.034; Fig. 3C). Estimates based on aggression and Bayesian clustering analysis of genetic data confirmed that neighboring nests of highland *L. turcicus* predominantly belong to different mutually aggressive and relatively small colonies, whereas all nests in *L. neglectus* populations belong to the same supercolony (Fig. 3D). This clearly indicates that all our sampled populations of *L. neglectus* are invasive, non-native populations expressing the full ‘invasive ant syndrome’. Lowland *L. turcicus* shows an intermediate social structure between the supercolonial invasive *L. neglectus* and the highly structured native highland *L. turcicus* populations: in the lowland *L. turcicus* populations, several ‘small-scale supercolonies’ coexist in most populations, but without coming close to the sizes of the extensive *L. neglectus* supercolonies (Fig. 3E).

### Reduced Variation of Recognition Cues

**Figure 4:**
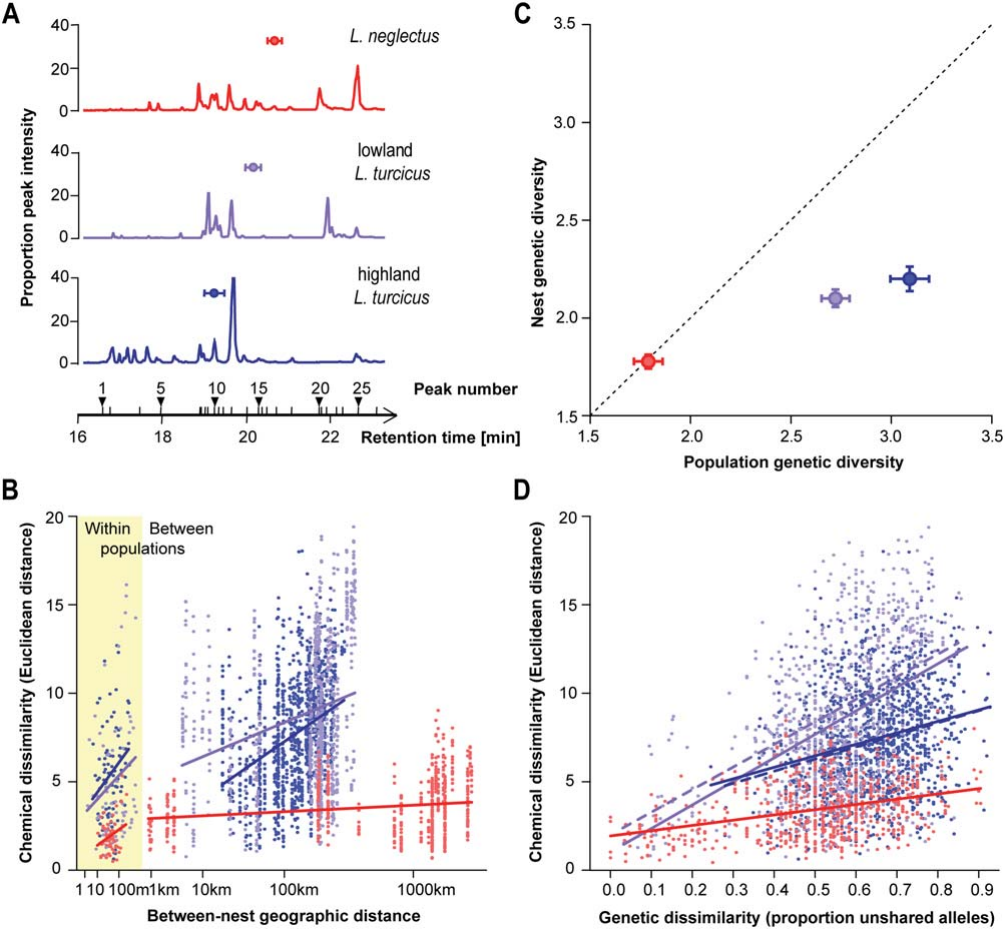
Variation in chemical recognition cues. (A) Typical cuticular hydrocarbon profiles of *L. neglectus* (red) and lowland/highland *L. turcicus* (pale and dark blue; referring to white, grey and black squares in Fig. 2A), with the 26 peaks (Table S2) plotted according to retention time. The relative contribution of long-chained versus short-chained hydrocarbons is indicated by the mean±s.e.m. weighted retention time. (B) Relationship between geographical distance and dissimilarity in cuticular hydrocarbons for each pair of nests. Least squares regression lines are shown separately for comparisons within and between populations. (C) Genetic diversity (mean allelic richness±s.e.m.) of nests and populations. (D) Relationship between genetic dissimilarity and dissimilarity in cuticular hydrocarbons for each pair of nests. Least squares regression lines are shown. For *L. turcicus*, dashed regression lines are also given for comparisons between pairs of nests that are within the range of within-nest allelic richness of *L. neglectus*.

Reduced efficiency of nestmate recognition has been hypothesized to enhance ant invasiveness, as lower degrees of antagonism facilitate the formation of large supercolonies. This has been suggested to happen if genetic variation at loci coding for cuticular hydrocarbons, the chemical recognition cues of social insects [35], would be reduced either by a genetic bottleneck during introduction [36] or by selection following introduction [10]. We therefore analyzed the properties of worker chemical profiles, and the correlations between profile variation among nests and populations on one hand, and geographical distance and genotype divergence at neutral genetic markers on the other. We found that the total quantity of cuticular hydrocarbons was similar across the three groups (Kruskal-Wallis test, *H* = 2.60, *P* = 0.27), but that both the overall profile characteristics and their variation differed. First, long-chain hydrocarbons were most abundant in the profiles of *L. neglectus*, relatively frequent in lowland *L. turcicus*, and rare in highland *L. turcicus* (Fig. 4A; weighted peak retention times: ANOVA, *F*_2,41_ = 46.44, *P*<0.001; *P*<0.05 in posthoc Tukey tests of all pairwise comparisons). Long-chain hydrocarbons are less volatile and have therefore been hypothesized to be less informative as recognition cues [37], which is consistent with the lower aggression levels across *L. neglectus* and (partly) lowland *L. turcicus*. Second, nests of *L. neglectus* were chemically much more similar to each other, both within and between populations, than nests of *L. turcicus* (Fig. 4B), which tend to belong to multiple independent colonies in each population (Figs. 3E, 4C). Within populations, differences in chemical profiles always increased with distance between nests (Fig. 4B, shaded area; Mantel test, all *P*<0.001), but differences in *L. neglectus* were small relative to those in *L. turcicus*. Among populations, however, differences remained low and did not increase significantly with distance for *L. neglectus* (Mantel test, *P* = 0.073), while both forms of *L. turcicus* showed significant increases of chemical cue dissimilarity with distance (Mantel tests: *P*<0.005), despite a smaller geographic sampling range.

Even if founder effects and subsequent genetic drift have reduced allelic richness across neutral marker loci in nests of *L. neglectus* relative to both forms of *L. turcicus* (Fig. 4C; 1.8 *vs.* 2.1 alleles on average in lowland populations and 1.8 *vs.* 2.2 in highland populations; ANOVA, *F*_2,198_ = 21.2, *P*<0.0001; *P*<0.001 for both comparisons in posthoc Tukey tests), this did not lead to a strongly reduced range of overall genetic dissimilarity across populations in *L. neglectus* (Fig. 4D). Differences in chemical profile increased with increasing genetic differentiation for both *L. neglectus* and the two forms of *L. turcicus* (Fig. 4D; Mantel tests, all *P*<0.001), but at every appreciable level of genetic differentiation, the level of chemical dissimilarity in *L. neglectus* was significantly lower than in *L. turcicus* (Fig. 4D; tested at the mean level of genetic differentiation of *L. neglectus*: *L. neglectus vs.* highland *L. turcicus*: Welch's *t*_13_ = 4.21, *P* = 0.001; *L. neglectus vs.* lowland *L. turcicus, t*_19_ = 5.40, *P*<0.001; highland *vs.* lowland *L. turcicus*: *t*_13_ = 1.20, *P* = 0.252). To ensure that this effect was not due to the somewhat lower allelic richness in colonies of *L. neglectus*, the analysis was repeated with the subset of *L. turcicus* nests that fell within the range of allelic diversities shown by *L. neglectus*, which gave the same result (Fig. 4D, dashed lines; Table S4). This implies that although some coupling between chemical profile variation and variation in neutral genetic markers has been maintained, the overall genetic component of chemical recognition cues in *L. neglectus* is considerably lower than in the non-invasive sister species. This low variation is consistently found across different introduction events [27], clearly suggesting that it was already present before introduction of *L. neglectus* outside the native range.

We conclude that *L. neglectus* expresses the full syndrome of traits that are known from other invasive ants, even though its introduction is relatively recent, and that there are multiple indications that these traits must have existed as pre-adaptations prior to invasion. Many of the same pre-adaptations to invasiveness were also detected in the lowland form of *L. turcicus*, which underlines that multiple components of the invasive garden ant syndrome may have provided significant selective advantages long before one of these species became invasive.

### Parasite Release and Invasive Success

Apart from factors related to social organization, gene flow and recognition that predispose certain ants to become invasive pests, ecological factors are most likely to determine which introductions will ultimately give rise to invasive range expansions. Many invasive species appear to owe their ecological success at least partly to release from parasites, as not all natural enemies follow their host into a new range when founder populations are small [38], [39], and alternative parasites from the new range might need time to adapt to a new host [40]. Introduced species are therefore expected to reduce investments in costly defense mechanisms that trade-off with growth and reproduction, as changes in this direction will provide significant advantages in competition with native species [41]. To test whether parasite release may have contributed to the abundance and spread of invasive garden ants, we analyzed the prevalence of two fitness-reducing micro-organisms: the intracellular reproductive parasite *Wolbachia*, which has been shown to occur frequently in native but not introduced populations of Argentine ants and fire ants [42]–[46], and the generalist fungal pathogen *Beauveria,* which is a common parasite of insects [47].

**Figure 5:**
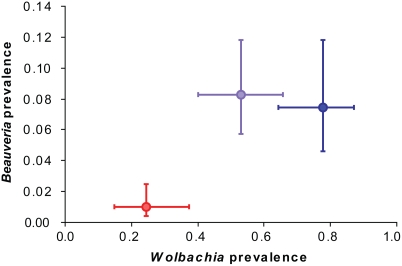
Parasite release. Prevalence (mean±s.e.m.) of the bacterium *Wolbachia* (*n* = 143 individuals, 30 populations) and the fungus *Beauveria bassiana* (*n* = 881 individuals, 27 populations) in *L. neglectus* (red) and lowland/highland *L. turcicus* (pale and dark blue).

Overall, there was a highly significant difference in parasite prevalence at the population level between *L. neglectus* and the two forms of *L. turcicus* (Fig. 5; logistic regression with parasite and ant group as factors, χ^2^ = 15.9, d.f. = 2, *P*<0.001). As the interaction term was not significant (χ^2^ = 1.68, d.f. = 2, *P* = 0.43) it appears that this difference in prevalence does not depend on the type of parasite. When analyzing the parasites separately, prevalence was significantly lower in *L. neglectus* compared to both forms of *L. turcicus* (Fig. 5; linear contrast, *Wolbachia*: χ^2^ = 9.60, d.f. = 1, *P* = 0.002, *Beauveria*: χ^2^ = 6.62, d.f. = 1, *P* = 0.01). These effects of major parasite release are likely to be most powerful shortly after introduction, as the positive effects will diminish when local parasites become adapted to a new host.

### The punctuated Evolution of Ant Supercolonies

Based on our analyses, we suggest that the evolution of supercoloniality in garden ants has been a multi-step process starting in the native Asia Minor range. It most likely began with the evolution of facultative mating within the nest, readoption of some newly-mated queens, and nest budding, leading to the extended family colonies that are typical for extant *L. turcicus* in lowland populations. The next crucial steps would then have been the changeover to a recognition system based on less volatile and less genetically variable cuticular hydrocarbons, creating single supercolonies per patch, and concomitant selection for reduced body size and flying ability of queens and males. A process of positive frequency dependent selection for dominant cuticular hydrocarbon profiles [10] may have been instrumental in achieving this result. The clear species status of *L. neglectus* and the relatively short history of invasiveness suggest that the substantial morphological changes in the queens and males also occurred in the native range long before invasive range expansion. This implies that the currently undiscovered native, non-invasive *L. neglectus* is expected to still occur somewhere in West Asia unless it recently went extinct. If this scenario is correct, human transport and parasite release [38], [39] would then have been sufficient conditions for the emergence of the large-scale supercolonies that characterize the extant invasive populations.

Our present report is the first to address all previously identified aspects of the evolution of ant invasiveness for a novel pest ant in a single multifaceted study. Our results show that invasive *L. neglectus* populations are a potential problem of global dimensions, and a particular threat for man-made ecosystems in the cold-temperate climate zones that have so far suffered very little from invasive ants. Although we have been able to answer many questions, major challenges remain. Future studies on the *L. neglectus*/*turcicus* model system should address when speciation events in the garden ant lineage took place and connect them to episodes of Pliocene/Pleistocene climate change. More extensive sampling in the Black Sea and Caspian Sea area would clarify much of the remaining *terra incognita* on the present distribution maps, and may allow the discovery of the non-invasive *L. neglectus* populations that were the source of the recent invasions in Europe and Asia. Only the discovery of native *L. neglectus* populations will ultimately allow us to determine the sequence of evolutionary transitions that our present study has inferred.

## Materials and Methods

### Ant Population Sampling

Live workers and sexuals were sampled from 43 *L. neglectus* (*Ln*) and *L. turcicus* (*Lt*) populations between 2003 and 2005 as detailed in the supporting information (Methods S1). Coordinates of the non-studied populations (Fig. 1B) were obtained from http://www.creaf.uab.es/xeg/Lasius/ and from unpublished data (notebooks B. Seifert).

### Behavioral observations

Aggression tests [27] between individual workers (on average four nests/population, 40 populations, 528 tests, 261 independent 1∶1 encounters, two replicates on average) were used to calculate population-specific aggression probabilities (the proportion of between-nest encounters that escalated into fighting), and the size of multiple-nest colonies (maximum distance between non-aggressive nests/population). We performed 270 mating experiments (163 pairings within and 107 pairings between the three ant forms) with virgin queens and males (9 populations; Methods S2).

### Genetic analyses

Microsatellite analysis at six loci ([27]; >30 workers from five nests/population on average, total 1362 individuals, 43 populations) provided a phylogeny of all sampled *Ln* and *Lt* populations with *L. austriacus* [32] as outgroup. The same data also produced estimates of nestmate relatedness, allelic richness per nest, between-nest genetic differentiation, and the size of multiple-nest colonies (maximum distance between nests that clustered in Bayesian analysis). An 1171 bp sequence of the mtDNA gene Cytochrome Oxidase 1 (73 workers, 1–4 nests/population, 42 populations) was used to obtain a Neighbor-Joining tree (Methods S3).

### Chemical analyses

Gas chromatography-mass spectrometry analyses ([27]; 5 pooled workers/nest, on average five nests/population, total 204 nests, 42 populations) revealed 26 cuticular hydrocarbons. An internal standard (C12) allowed determination of the overall quantity of the hydrocarbons for each sample. Peak retention times and areas were used to calculate weighted overall retention times for each profile. Chemical dissimilarities were subsequently calculated as Euclidean distances between the first six components of a Principal Component Analysis of peak areas (Methods S4).

### Morphology

Fourteen morphological parameters (Methods S5) were measured for three to five workers per nest (144 nests, 43 populations). Only head size was measured for the sampled queens (*n* = 93) and males (*n* = 42; 22 populations for both).

### Comparison of distance measures

The relationship between chemical, genetic and geographical distances between pairs of nests within each of the three forms was investigated using a modified Mantel test in which within- and between-population relationships were examined separately. Geographical distances were Box-Cox transformed to normalize their distribution and to reduce biases in within- versus between-population effects. Regression slopes from these pairwise analyses, predicted values of the dependent variable, within- and between-population variances in the dependent variable, and standard errors were all estimated by jack-knifing over populations (Methods S6).

### Parasite prevalence

The prevalence of field infections with the intracellular bacterium *Wolbachia* was tested (on average five nests/population, 143 nests, 30 populations). We first performed conventional PCR amplification using the *wsp* gene of *Wolbachia* [42], and then additionally carried out high-resolution quantitative PCR of the *ftsZ* gene (Roche 2.0 LightCycler and SYBR green; Roche Biochemicals; [48]) for samples for which no infection could be scored. The prevalence of the entomopathogenic fungus *Beauveria bassiana* was determined by inspection of worker corpses (>30 workers/population, 881 individuals, 27 populations) that had been kept in a humid chamber at 23°C for 20 days after death (Methods S7). Differences in population prevalence between species were tested with a logistic regression model with binomial error structure applying the GENMOD procedure in SAS/enterprise while estimating the scaling parameter by the square root of DEVIANCE/DOF.

### Statistical analyses

Assumptions for normality and homogeneity of variances were tested and necessary transformations performed before applying parametric statistical tests in JMP 7.02, Statistica 6, Sigma Stat 2.03, SPSS 10 or SAS/enterprise. Reported *P* values are two tailed.

## Supporting Information

Figure S1 Distribution maps including population numbers for L. neglectus (N1-18) and L. turcicus (T1-25; see also Fig. 1 and table S1).
(2.33 MB TIF)

Figure S2 The microsatellite consensus tree of Fig. 2A, including population information and bootstrap values >50% for all except terminal nodes. For population identification see Fig. S1 and table S1.
(1.16 MB TIF)

Figure S3 Neighbor Joining (NJ) tree of CO1-haplotypes with bootstrap values (above the branch; see Fig. 2A) and additional posterior probabilities (Bayesian Markov Chain Monte Carlo; below the branch in italics) for all except terminal nodes. For each sample, population ID (see Fig. S1 and table S1), nest identification number (in brackets), as well as GenBank identification number are given.
(1.61 MB TIF)

Methods S1 Ant Sampling and Identification of Populations
(0.03 MB DOC)

Methods S2 Behavioral Observations
(0.03 MB DOC)

Methods S3 Genetic Analysis
(0.04 MB DOC)

Methods S4 Chemical Analysis of Cuticular Hydrocarbons
(0.04 MB DOC)

Methods S5 Morphology
(0.05 MB DOC)

Methods S6 Comparison of Geographical, Genetic and Chemical Distances
(0.03 MB DOC)

Methods S7 Parasite Prevalence
(0.03 MB DOC)

Table S1 Population localities: countries, names, coordinates and altitudes of the populations.
(0.14 MB DOC)

Table S2 List of cuticular hydrocarbon compounds and their mean proportional peak areas in the three profile types (coded as white, grey or black squares in Fig. 2A)
(0.09 MB DOC)

Table S3 Morphological parameters measured for workers of L. neglectus, lowland and highland L. turcicus (mean±s.d. of measurements of the three groups, derived from population means, n = 18, 12, and 13 populations).
(0.06 MB DOC)

Table S4 Summary of regression analyses of chemical dissimilarity as a function of geographical distance and genetic dissimilarity.
(0.10 MB DOC)
