# Supplementary figures and images for: The Evolution of Invasiveness in Garden Ants
Source: PLoS One. 2008 Dec 3;3(12):e3838. doi: 10.1371/journal.pone.0003838 (PMC2585788; doi:10.1371/journal.pone.0003838)

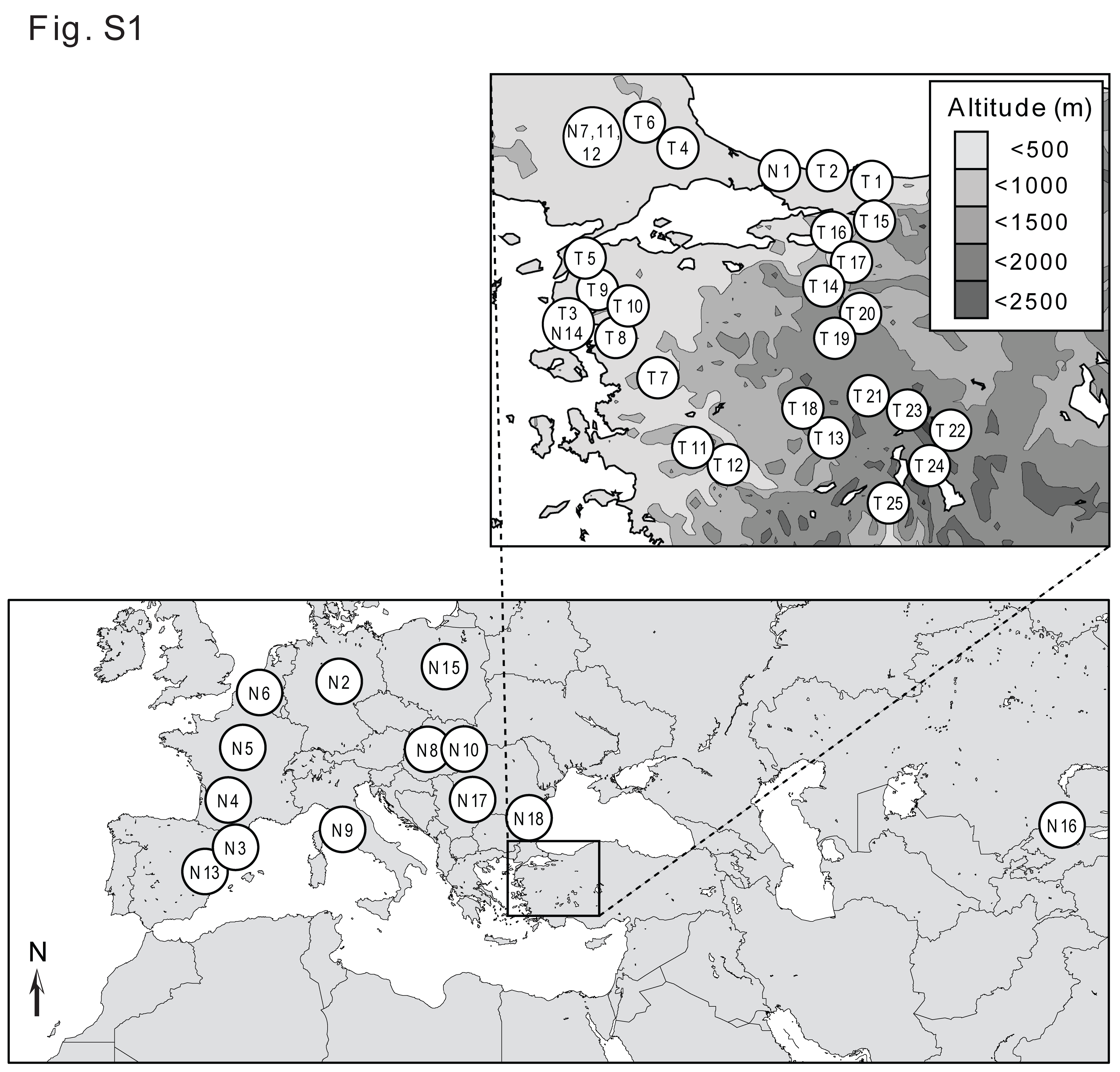

Supplement: Figure S1 — Distribution maps including population numbers for L. neglectus (N1-18) and L. turcicus (T1-25; see also Fig. 1 and table S1). (2.33 MB TIF) [file pone.0003838.s001.tif]

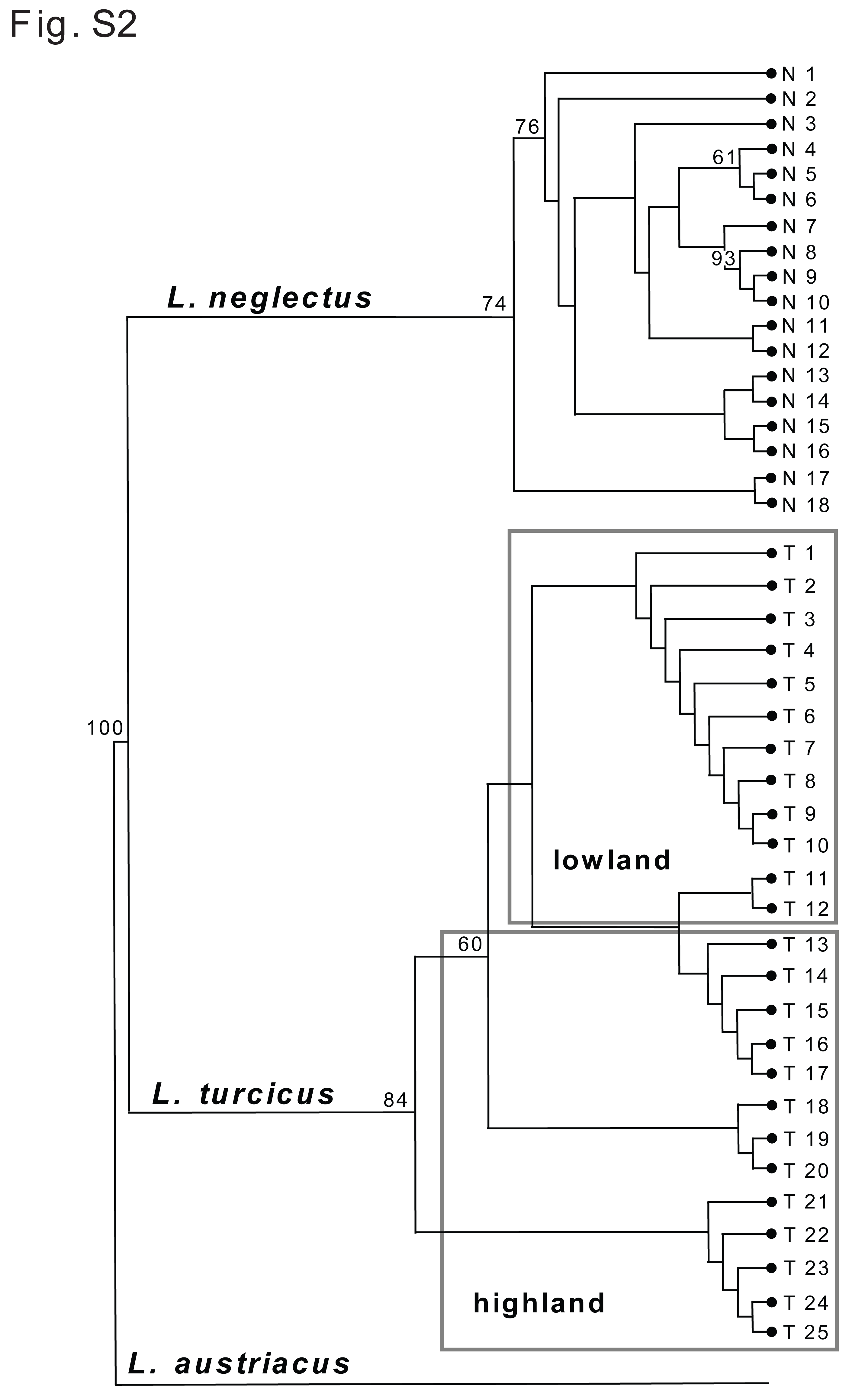

Supplement: Figure S2 — The microsatellite consensus tree of Fig. 2A, including population information and bootstrap values >50% for all except terminal nodes. For population identification see Fig. S1 and table S1. (1.16 MB TIF) [file pone.0003838.s002.tif]

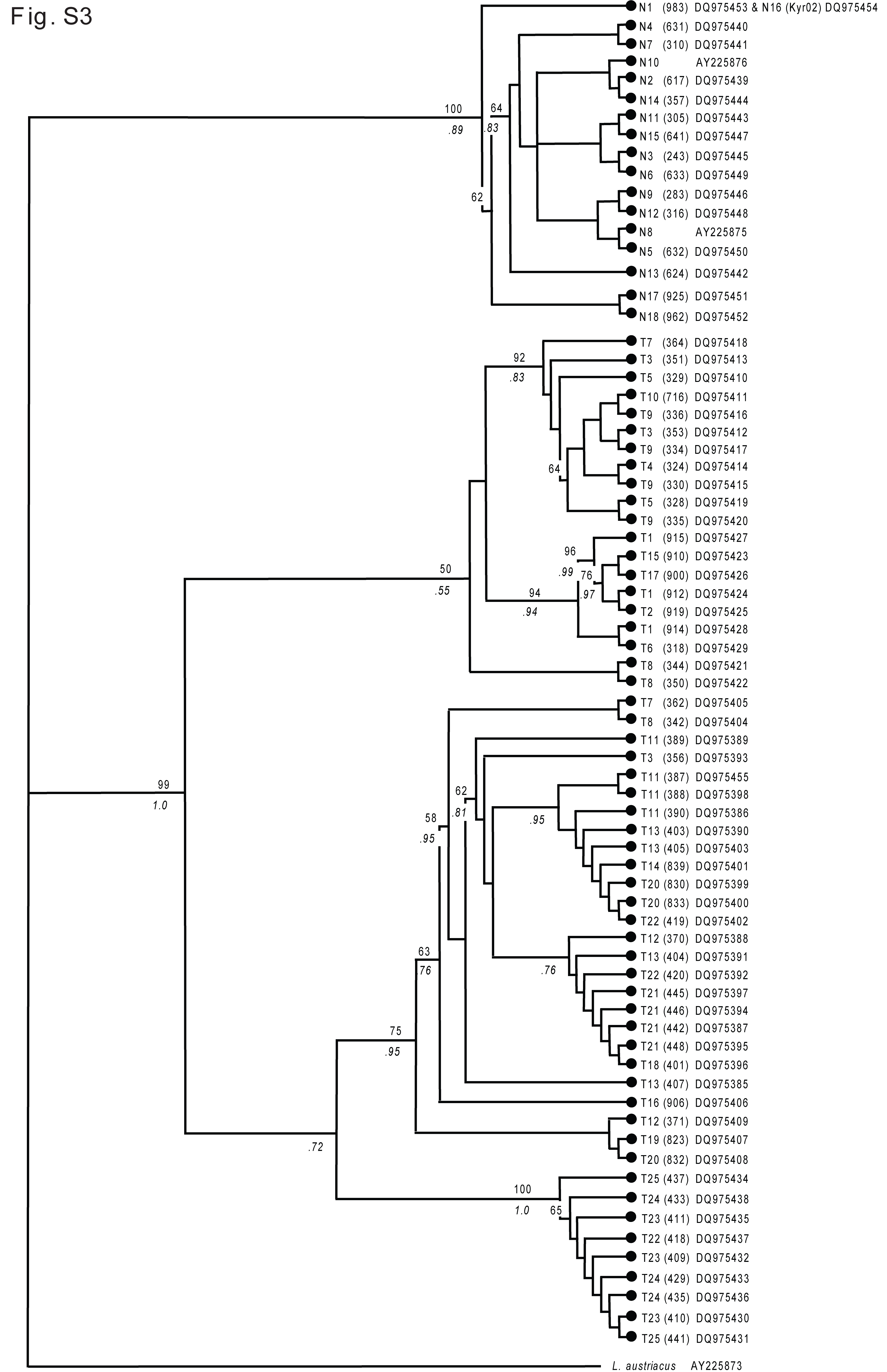

Supplement: Figure S3 — Neighbor Joining (NJ) tree of CO1-haplotypes with bootstrap values (above the branch; see Fig. 2A) and additional posterior probabilities (Bayesian Markov Chain Monte Carlo; below the branch in italics) for all except terminal nodes. For each sample, population ID (see Fig. S1 and table S1), nest identification number (in brackets), as well as GenBank identification number are given. (1.61 MB TIF) [file pone.0003838.s003.tif]
