# Supplementary material for: The Evolution of Invasiveness in Garden Ants
Source: PLoS One. 2008 Dec 3;3(12):e3838. doi: 10.1371/journal.pone.0003838 (PMC2585788; doi:10.1371/journal.pone.0003838)
Supplement: Methods S1 — Ant Sampling and Identification of Populations (0.03 MB DOC) [file pone.0003838.s004.doc]

# Methods S1

## Ant Sampling and Identification of Populations

We sampled 50–200 workers from 2–14 nests (mean 4.8 ± 1.9 s.d.) in 18 populations of *L. neglectus* (*Ln*) and 25 populations of *L. turcicus* (*Lt*) in May 2003 and June 2004 (Fig. 1). In June 2005 we were also able to collect 1–35 reproducing queens from 100 of these nests in 17 populations. *L. austriacus* samples (four nests) were collected from a population in Austria [S1] in August 2002. Population identification numbers and coordinates of sampled populations are given in Fig. S1 and Table S1. Coordinates from the non-studied populations shown in Fig. 1B were obtained from http://www.creaf.uab.es/xeg/Lasius/ and from unpublished data of B.S.

## References

S1. Steiner FM, Schlick-Steiner BC, Schödl S, Espadaler X, Seifert B, et al. (2004) Phylogeny and bionomics of *Lasius austriacus* (Hymenoptera, Formicidae). Insect Soc 51: 24-29.
