# Supplementary material for: The Evolution of Invasiveness in Garden Ants
Source: PLoS One. 2008 Dec 3;3(12):e3838. doi: 10.1371/journal.pone.0003838 (PMC2585788; doi:10.1371/journal.pone.0003838)
Supplement: Methods S2 — Behavioral Observations (0.03 MB DOC) [file pone.0003838.s005.doc]

# Methods S2

## Behavioral Observations

*Aggression Tests*

Aggression tests were performed in the laboratory between individual workers of different nests of the same population (mean 4.3 ± s.d. 0.8 nests/population) in all but three (N1, N16, T15) populations (16 populations of *L. neglectus*, 130 independent encounters; 12 populations of lowland *L. turcicus*, 62 independent encounters; 12 populations of highland *L. turcicus*, 69 independent encounters; total 528 aggression tests, mean 13 replicates per population). Five minutes before the test, individual ants were removed from their rearing boxes, placed in an individual plastic vial (diameter 2 cm, height 5 cm; fluon™ coated sides). The ants were then placed individually into the ‘fighting arena’ (Petri dish, 5.5 cm diameter; preliminary tests revealed that the outcome of the fight was independent of the order of putting the ants into the dish), and observed for 10 min at an ambient temperature of 22.5 ± 2°C. The frequency of six behaviors [ignorance behavior, antennation, avoidance behavior, gaster (abdomen) raising for spraying with formic acid, biting, fighting; as defined in Giraud *et al.* [S2]] was recorded as described in Ugelvig *et al.* [S3]. The mean proportion of all encounters in which aggression was shown (gaster raising, biting or fighting) was determined per nest pair, and the population mean was calculated as the mean of these proportions for all the nest pairs of the particular population, revealing the population-specific aggression probability. The extent of multiple-nest colonies was estimated as the maximum distance between non-aggressive nests for each population.

*Mating Experiments*

In June 2005, virgin (winged) queens and males were collected in nine of the study populations within their natal nests (2 *L. neglectus*, N11 and N14; 3 lowland *L. turcicus* populations, T6, T7, T10; and 4 highland *L. turcicus* populations T13, T20, T23, T24). Directly after collection, mating experiments were conducted in the field, where 1 queen and 1 male of the same form were placed together in a plastic box (7.5 × 5.5 × 5 cm) with transparent lid (*L. neglectus*: *n* = 27 pairings; lowland *L. turcicus*: *n* = 30 pairings; highland *L. turcicus*: *n* = 106 pairings), and observed for any mating attempts over the course of four hours (scanned every 15–30 min). In addition, 107 pairings were conducted between the three forms (*L. neglectus* *vs.* lowland *L. turcicus*: *n* = 12 pairings; *L. neglectus* *vs.* highland *L. turcicus*: *n* = 15 pairings; lowland *vs.* highland *L. turcicus*: *n* = 80 pairings).

Frequencies were compared between groups using χ^2^ tests. Calculations of the 95% confidence intervals for binomial distributions were performed following Zar [[S4] Section 24.4, pp. 527–530].

## References

S2. Giraud T, Pedersen JS, Keller L (2002) Evolution of supercolonies: the Argentine ants of southern Europe. P Natl Acad Sci USA 99: 6075-6079.

S3. Ugelvig LV, Drijfhout FP, Kronauer DJC, Boomsma JJ, Pedersen JS, et al. (2008) The introduction history of invasive garden ants in Europe: integrating genetic, chemical and behavioural approaches. BMC Biol 6: 11.

S4. Zar JH (1999) Biostatistical Analysis. New Jersey: Prentice Hall.
