# Supplementary material for: The Evolution of Invasiveness in Garden Ants
Source: PLoS One. 2008 Dec 3;3(12):e3838. doi: 10.1371/journal.pone.0003838 (PMC2585788; doi:10.1371/journal.pone.0003838)
Supplement: Methods S3 — Genetic Analysis (0.04 MB DOC) [file pone.0003838.s006.doc]

# Methods S3

## Genetic Analysis

## 3a. Microsatellite Data

*Sampling and DNA Extraction*

DNA was extracted from an average of 31.7 workers (range 24–36) from each of the 43 populations of *L. neglectus* and *L. turcicus*. Where possible (in 36 populations), these workers were collected from at least 5 different nests [overall mean nest number per population was 4.8 ± 1.9; total of 1362 individuals from 206 different nests, of which 420 individuals from 14 *Ln* populations were also used in a previous study [S3]]. In addition, DNA was extracted from 31 individuals from four nests of one population of *L. austriacus*. In all cases the mesosoma (thorax) was crushed in 200 µl of 5% Chelex solution and boiled for 15 min at 99°C.

*Primers and PCR Conditions*

Six polymorphic microsatellite loci were used with the PCR conditions described by Ugelvig *et al.* [S3]. All loci worked equally well for *L. neglectus* and *L. turcicus,* whereas one locus (Lng1) did not amplify in *L. austriacus*. Analysis was performed with Genescan 3.1 and Genotyper 3.1 (PE-Applied Biosystems).

*Tree Construction*

A phylogenetic tree (consensus tree, Figs. 2A and S2) was created based on a distance matrix of allele frequency data (following the ‘additive tree model’) with the program Phylip 3.6 [[http://evolution.genetics.washington.edu/phylip.html](https://frontserver.bignet.ku.dk/exchweb/bin/redir.asp?URL=http://evolution.genetics.washington.edu/phylip.html); [S5]]; from (1) all six microsatellite loci for all samples of *L. neglectus* and *L. turcicus*, and (2) including *L. austriacus* as an outgroup from the five microsatellite loci that amplified across all three species. Genetic distances were calculated using Cavalli-Sforza chord measures. Bootstrap values were obtained by 2000 permutations and are given at all but the terminal nodes when >50% (see Fig. 2A). For the *L. neglectus* and *L. turcicus* clades, the use of five rather than six microsatellite loci led to no supported changes in the trees.

*Population Genetic Analysis*

All six microsatellite loci were used for the analysis of genetic population structure. FSTAT 2.9.3.2 [http://www2.unil.ch/popgen/softwares/fstat.htm; [S6]] was used to calculate relatedness among nestmates and allelic richness (Fig. 3C) per nest (i.e. the number of alleles per locus, corrected for differences in sample size using rarefaction to estimate the allelic richness expected for 3 individuals per nest). Nestmate regression relatedness was obtained per nest based on the allelic distribution in the entire population. Genetic dissimilarity between pairs of nests (Fig. 4D) was calculated as the proportion of all possible alleles that were unique to either nest (i.e. if all alleles present in one nest were also present in another and *vice versa*, genetic dissimilarity was zero, if no alleles were shared between nests, genetic dissimilarity was one). This allele-based measure was chosen as it gives a measure of the genetic dissimilarity between two nests without the pseudoreplication inherent in using individual-based measures of genetic dissimilarity when nests are made up of relatives, as is expected in *Lasius* ants.

*Cluster Analysis*

The Bayesian clustering program BAPS 2.0 [http://www.abo.fi/fak/mnf/mate/jc/ smack_software_eng.html; [S7]]; was used to determine nests that cluster together in groups with high genetic similarity. The sampling level was ‘population’ with ‘nests’ as a sub-level. Enumerative calculations were used for estimating the population structure when the number of nests was less than nine, which was the case in all but one population, T22 (*n* = 14). In this single case estimation of population structure was performed by a Markov Chain Monte Carlo (MCMC) approach using 50,000 iterations with thinning = 3 and a burn-in period of 10,000.

## 3b. mtDNA Sequencing

*Samples*

One individual per population was sequenced for each of the 18 *L. neglectus* populations, except for the two Hungarian populations, for which sequences have already been published [[S1]; N8: AY225875 and N10: AY225876]. For the 25 *L. turcicus* populations, one to four individuals from different nests of the population were sequenced (see Figs. 2A and S3; total: 56 samples, mean 2.25 nests/population).

*Sequencing*

DNA extractions and PCR conditions for amplification of the Cytochrome Oxidase 1 (CO1) gene followed the protocols of Steiner *et al.* [S8]. Primers used for PCR were the unpublished LNEGF (5'-TAATGATGGAGTYGGAACAGG-3') combined with L2-N-3014r alias ‘Pat’ [S9]. For sequencing we used Pat and the unpublished internal primer LNEGR (5'-ATAAATCCTATTGYTCATCATA-3'). For all samples except one (population N16, for which only 450 bp could be sequenced), we obtained a sequence of 1171 bp. Sequences are deposited at GenBank with accession numbers DQ975385–DQ975455 (see Fig. S3 for details).

*Sequence Alignment and Tree Construction*

Sequence alignment was performed with the default settings of Clustal X [ftp://ftp-igbmc.u-strasbg.fr/pub/ClustalX/; [S10]]. A published sequence of *L. austriacus* [[S1]; AY225873], the next relative of *L. neglectus* and *L. turcicus*, was taken as outgroup. For tree construction, Neighbor Joining (NJ) based on Tamura-Nei distance [S11] and Bayesian Markov Chain Monte Carlo (BMCMC) phylogenetic analyses were performed using Mega 2 [http://www.megasoftware.net/; [S12]] and MrBayes 3.1.2 [http://mrbayes.csit.fsu.edu/; [S13]], respectively. 1000 bootstrapping replicates were performed on the NJ tree. Prior to BMCMC analysis the best-fitting nucleotide substitution model (GTR+G) was selected by using the hierarchical likelihood ratio test [S14] implemented in MrModeltest 2.2 [http://www.csit.fsu.edu/~nylander/; [S15]]. In the BMCMC analyses we performed two runs with default settings (one cold and three heated chains; heating temperature = 0.2, sampling frequency = 100) for 1.5 × 10^6^ generations, with the model parameters being estimated during the analysis. As the standard deviations of split frequencies always remained below 0.01 after ca. 10^6^ generations, we used the last 5000 trees to compute a majority-rule consensus tree and the posterior probabilities for nodes.

*Testing of Alternative Trees*

To test the robustness of our obtained tree, in which *L. neglectus* appeared as a clearly separated clade from *L. turcicus*, MrBayes 3.1.2 was also used to run MCMC analyses with the *a priori* constraint of the 20 haplotypes from the pure lowland *L. turcicus* clade (haplotypes 318, 324, 328, 329, 330, 334, 335, 336, 344, 350, 351, 353, 364, 716, 900, 910, 912, 914, 915, and 919) being monophyletic with *L. neglectus* (1.2 × 10^6^ generations). Convergence was achieved after 9 × 10^5^ generations (standard deviations of split frequencies were always <0.008). Thus the last 3000 trees were used to construct the majority rule consensus tree. For a comparison of the unconstrained versus constrained trees we used the SH-test [S16] with RELL (resampling estimated log-likelihood) estimates of the test distribution. The SH-test indicated that the tree in which the 20 haplotypes of lowland *L. turcicus* were forced to be monophyletic with *L. neglectus* is significantly worse than the unconstrained tree (*P* = 0.012).

## References

S1. Steiner FM, Schlick-Steiner BC, Schödl S, Espadaler X, Seifert B, et al. (2004) Phylogeny and bionomics of *Lasius austriacus* (Hymenoptera, Formicidae). Insect Soc 51: 24-29.

S3. Ugelvig LV, Drijfhout FP, Kronauer DJC, Boomsma JJ, Pedersen JS, et al. (2008) The introduction history of invasive garden ants in Europe: integrating genetic, chemical and behavioural approaches. BMC Biol 6: 11.

S5. Felsenstein J (1989) PHYLIP - Phylogeny Inference Package (Version 3.2). Cladistics 5: 164-166.

S6. Goudet J (1995) FSTAT (version 1.2): a computer program to calculate F-statistics. J Hered 86: 485-486.

S7. Corander J, Waldmann P, Marttinen P, Sillanpaa MJ (2004) BAPS 2: enhanced possibilities for the analysis of genetic population structure. Bioinformatics 20: 2363-2369.

S8. Steiner FM, Schlick-Steiner BC, Sanetra M, Ljubomirov T, Antonova V, et al. (2005) Towards DNA-aided biogeography: an example from *Tetramorium* ants (Hymenoptera, Formicidae). Ann Zool Fenn 42: 23-35.

S9. Simon C, Frati F, Beckenbach A, Crespi B, Liu H, et al. (1994) Evolution, weighting, and phylogenetic utility of mitochondrial gene sequences and a compilation of conserved polymerase chain reaction primers. Ann Ent Soc Am 87: 651-701.

S10. Thompson JD, Gibson TJ, Plewniak F, Jeanmougin F, Higgins DG (1997) The Clustal-X windows interface: Flexible strategies for multiple sequence alignment aided by quality.

S11. Tamura K, Nei M (1993) Estimation of the number of nucleotide substitutions in the control region of mitochondrial DNA in humans and chimpanzees. Mol Biol Evol 10: 512-526.

S12. Kumar S, Tamura K, Jakobsen IB, Nei M (2001) MEGA 2: molecular evolutionary genetics analysis software.

S13. Ronquist F, Huelsenbeck JP (2003) MrBayes 3: Bayesian phylogenetic inference under mixed models. Bioinformatics 19: 1572-1574.

S14. Huelsenbeck JP, Rannala B (1997) Phylogenetic methods come of age: Testing hypotheses in a phylogenetic context. Science 276: 227-232.

S15. Nylander JAA (2004) MrModeltest v2. Uppsala University: Program distributed by the author. Evolutionary Biology Centre.

S16. Shimodaira H, Hasegawa M (1999) Multiple comparisons of log-likelihoods with applications to phylogenetic inference. Mol Biol Evol 16: 1114-1116.
