# Supplementary material for: The Evolution of Invasiveness in Garden Ants
Source: PLoS One. 2008 Dec 3;3(12):e3838. doi: 10.1371/journal.pone.0003838 (PMC2585788; doi:10.1371/journal.pone.0003838)
Supplement: Methods S4 — Chemical Analysis of Cuticular Hydrocarbons (0.04 MB DOC) [file pone.0003838.s007.doc]

# Methods S4

## Chemical Analysis of Cuticular Hydrocarbons

*Sampling and Extraction*

We sampled 204 nests from 42 populations (*L. neglectus*, *n* = 77 nests from 17 populations only, since no live material was available from population N1; lowland *L. turcicus*: *n* = 64 and highland *L. turcicus*: *n* = 63 nests; 4.9 ± 2.0 nests per population). Immediately after transport of the live ants into the laboratory, five ants per nest were placed together in glass vials (Supelco; 1.8 ml; 3–5 replicates each), and stored at –20°C until solvent extraction. Extraction of cuticular compounds with pentane was performed as described in Ugelvig *et al*. [S3]. Extracts were dried and stored sealed at –20°C until they were analyzed on a Gas Chromatograph, coupled to a Mass Spectrometer (GC-MS).

*Gas Chromatography – Mass Spectrometry*

Shortly before GC-MS analysis, the samples were re-diluted in 20 µl of hexane containing an internal standard (dodecane, C12) at a concentration of 2.5 pg/µl to allow quantitative analysis. 2 µl of the extract was injected splitless into the GC-MS (Agilent Technologies 6890N coupled to a 5973N MSD) equipped with a HP-5 column (30 m × 0.25 mm, 0.25 µm film thickness) following the procedure described in Ugelvig *et al.* [S3].

*Analysis and Choice of Compounds*

Identification of hydrocarbons followed the procedures described in Ugelvig *et al.* [S3]. In the whole dataset of the three ant forms, 26 hydrocarbons were detected, which varied in chain length between C31 and C35 (for details see Table S2 and Fig. 4A). These 26 compounds are referred to as the cuticular hydrocarbons (CHC) of the analyzed samples.

*Year Effect*

The data from 2003 and 2004 were pooled in the statistical analysis, as the collecting year had no significant effect (General Linear Model on ‘population’ and ‘year’ as factors: populations: *F*_15,102_ = 59.8, *P*<0.001; year: *F*_1,102_ = 0.5, *P* = 0.482; interaction: *F*_15,102_ = 1.2 *P*_population-year_ = 0.290) in the 16 populations where samples were taken in both years.

*Qualitative and Quantitative Hydrocarbon Analysis*

The samples fell into three qualitative groups (see Figs. 2B and 4A), defined by e.g. a high peak (#25) in *L. neglectus*, a high double peak (#20 and 21) in lowland *L. turcicus* and a very high peak (#13) without significant amounts of any of the longer-chain hydrocarbons such as C35 in highland *L. turcicus* (see also Table S2).

For all 204 nest samples, the area below each peak was integrated and the relative proportions of the 26 hydrocarbon peaks used in a Principal Component Analysis PCA based on covariances (JMP 7.01) to reduce the number of variables and remove correlations between them for subsequent analysis. This produced six components that cumulatively explained 96.2% of the variation in peak areas of the original 26 compounds (explaining, respectively, 51.9, 28.1, 7.5, 4.6, 2.6, and 1.5% of the original variance). To quantitatively confirm the affiliation of each nest sample to the qualitative grouping made ‘by eye’, a discriminant analysis based on the principal components was run with the three profile types as grouping variable (Wilks’ λ = 0.0065, *F*_12,392_  = 372.9, *P* < 0.0001). All 77 *L. neglectus* samples had posterior probabilities of *P* = 1.0, whereas in *L. turcicus*, the posterior probabilities of 3% of the nests (4/127) from 4/25 populations (T1, T16, T17, T23) were below 0.95 (mean posterior probabilities of these nests 0.7). Our Discriminant Analysis of the quantitative peak intensities thus confirmed the qualitative group assignment, since, in total, 98% of the samples had posterior probabilities >0.95. As all nests of the same population could consistently be assigned to one of the three types, a single symbol is given per population in Fig. 2A (*L. neglectus:* white square; lowland *L. turcicus*: grey square, and highland *L. turcicus*: black square).

The internal standard was used to calculate the overall quantity of the cuticular hydrocarbons per peak for each nest sample, which was compared by ANOVA between the three groups. For each sample, the mean weighted retention time was calculated by multiplying the retention time of each peak by the intensity of that peak (given as the proportion of the whole) and then taking the sum of these values for all peaks of the profile. The mean ± s.e.m. of the weighted retention time was then calculated for each of the three ant groups (Fig. 4A). Chemical dissimilarity between each pair of nests within each group was calculated as the Euclidean distance between the profile of each nest, defined as the set of six principal component values for each nest.

## References

S3. Ugelvig LV, Drijfhout FP, Kronauer DJC, Boomsma JJ, Pedersen JS, et al. (2008) The introduction history of invasive garden ants in Europe: integrating genetic, chemical and behavioural approaches. BMC Biol 6: 11.
