# Supplementary material for: The Evolution of Invasiveness in Garden Ants
Source: PLoS One. 2008 Dec 3;3(12):e3838. doi: 10.1371/journal.pone.0003838 (PMC2585788; doi:10.1371/journal.pone.0003838)
Supplement: Methods S5 — Morphology (0.05 MB DOC) [file pone.0003838.s008.doc]

# Methods S5

## Morphology

*Samples*

Three to five workers of each of 144 nests from all the 43 populations (mean 3.6 ± 4.0 s.d. nests per population), were individually measured for 14 morphological parameters (see below). Sexuals (queens and males) could only be collected from 22/43 populations (10 populations of *L. neglectus*, 7 populations of the lowland and 5 of highland *L. turcicus* form, respectively: 42 individual male samples from 17 populations (8 *L. neglectus*: N3, N6, N8, N11, N13, N14, N16, and N17; 4 lowland *L. turcicus*: T5, T6, T7, and T10; 5 highland *L. turcicus*: T13, T14, T20, T23, and T24), and 93 individual queen samples from 18 populations (8 *L. neglectus*: N2, N3, N4, N6, N8, N11, N13, and N16; 6 lowland *L. turcicus*: T1, T2, T5, T7, T9, and T10; 4 highland *L. turcicus*: T13, T20, T23, and T24).

*Morphometric Measurements*

The following primary numerical characters [described in more detail in Seifert [S17,18] and Schlick-Steiner *et al.* [S19]] were obtained for each worker specimen:

1. Cephalic length: maximum head length along the median line.
2. Cephalic width: maximum head width across, behind, or before the eyes.
3. Scape length: maximum straight-line length of the first antennal segment (scape), excluding the articular condyle.
4. Distance clypeus-antennal sockets: shortest distance from posterior clypeal suture to the upper inner margin of the antennal sockets.
5. Eye-size index: the arithmetic mean of the large and small axis of the elliptical compound eye, including all structurally visible ommatidia (i.e. also including unpigmented ommatidia).
6. Postocular distance: the distance between the median point of the occipital margin and the median point of the projected connection line between the caudalmost points of the left and right eye [S20], measured in the same spatial adjustment as for cephalic length.
7. Length of pronotal setae: maximum length of setae (thick hairs) on the pronotum.
8. Length of gulal setae: maximum length of setae on the underside of the head (gula).
9. Number of gulal setae: number of setae on the gula as seen in full profile. Arithmetic mean of bilateral counts.
10. Number of hind-tibial setae: number of setae on the extensor profile of the hind tibia, excluding the apical setae. Arithmetic mean of bilateral counts.
11. Number of occipital setae: number of setae projecting from the hind margin of the vertex frontal to the caudal end of the eye (occiput). Arithmetic mean of bilateral counts.
12. Number of scape setae: number of setae on the dorsal plane of the scape, excluding the most apical (distal) setae. Arithmetic mean of bilateral counts.
13. Number of subspiracular setae: number of setae on the area between the lower margin of the propodeal spiracle and the upper margin of the bulla of the metapleural gland. Arithmetic mean of bilateral counts.
14. Pubescence: the square root of the distance between fine hairs on the clypeus.

Head size was calculated as the arithmetic mean of the head length and width, and was used as the only body size indicator for the samples of sexuals (93 queens and 42 males from 10 *Ln* and 12 *Lt* populations; see Fig. 3A). All other measurements are given after allometric correction [S19,21], assuming a head size of 0.9 mm for all specimens.

All measurements were performed on mounted and dried specimens using a Wild M10 high-performance stereomicroscope equipped with a 1.6 × planapochromatic objective (magnifications of 64-320 ×). A Schott KL 1500 cold-light source was used for measuring silhouette lines, whereas a Schott KL 2500 LCD cold-light source in combination with a Leica coaxial polarized-light illuminator provided optimum resolution of tiny structures and micro-sculpture at the highest magnifications. A Leica cross-scaled ocular micrometer with 120 graduation marks ranging over 65% of the visual field was used. To avoid parallax error, its measuring line was constantly kept vertical within the visual field. Measuring errors are ± 0.6 µm for small and well-defined structures such as eye size, but ± 3 µm for larger structures with humidity-induced and positioning errors [S21]. To avoid rounding errors, all measurements were recorded in µm even for characters for which a precision of ± 1 µm was impossible. Setae were differentiated from pubescence hairs in having a distinctly larger basal diameter. All seta counts (9–13) were restricted to standing setae projecting >20 µm from the silhouette of the cuticular surface as observed under transmitted-light, and are arithmetic means of the bilateral counts. Measurements of body parts always refer to the real cuticular surface and not to the diffuse pubescence surface.

*Species Determination and Statistical Analysis*

The mean of all measurements of individual workers from the same nest (3–5 workers each) was calculated for each character, and was further used to determine the species affiliation of the sample. Nests with very small workers that had a large distance between the clypeus and antennal sockets, a large number of occipital setae, and a small postocular distance were determined as *L. neglectus*. The grouping of nests into *L. neglectus* and the two altitudinal forms of *L. turcicus*, referred to as small (lowland) and large (highland) *L. turcicus* workers, was performed by binary discriminant analyses using qualitative CHC grouping as *a priori* hypotheses (details available on request). Zoological names were assigned by adding the type samples of *L. neglectus* (from Budapest, Hungary) and *L. turcicus* (from Ankara, Turkey) to the data set as zero-values (implying no *a priori* hypothesis on their identity). The DA resulted in a clear *a posteriori* allocation of the *L. turcicus* types to the highland form (*P* = 0.996) and of the *L. neglectus* types to the CHC cluster that we previously identified as *L. neglectus* (*P* = 1.000). The numeric MOBAT analysis [S22] thus established that only the highland population matches the syntype series of *L. turcicus* Santschi 1921. In 19/25 *L. turcicus* populations, all nest samples could consistently be assigned to either the small (lowland) or large (highland) worker type, while in the remaining six populations (T1, T2, T13, T19, T20, and T23), some nests were assigned to the small and others to the large worker size class. For these populations the proportion of small size class (grey sectors of circles) and large size class nests (black sectors) are shown in Fig. 2A. A total of 15 *L. turcicus* nest samples could not be assigned to their group with a probability of *P* > 0.95, and 12 of these fell into the above mentioned populations that consisted of nests with both small and large sized workers. These nests were included in the analysis as belonging to the group they supported most, as including or excluding these samples did not significantly alter the proportions of small and large nests in the 6 populations: Wilcoxon sign rank test; *W* = 0.000, *P* = 1.0). The observed variation of worker sizes within populations might be caused by sampling colonies of different age, since young colonies may not yet have the resources to develop ‘normal sized’ workers, but produce much smaller workers [S23]. Alternatively, they could reflect some degree of hybridization between the two forms of *L. turcicus*.

Table S3 summarizes the morphological measurements for the three groups, taking only those populations for which all nest samples fell into the same morphological group into account, (all 18 populations of *L. neglectus*, 10/12 lowland populations of *L. turcicus*, 9/13 highland populations of *L. turcicus*, see above and Fig. 2A).

## References

S17. Seifert B (1992) A taxonomic revision of the palearctic members of the ant subgenus *Lasius* s. str. (Hymenoptera: Formicidae). Abh Ber Naturkundemus Goerlitz 6: 1-67.

S18. Seifert B (1996) Ameisen beobachten, bestimmen. Augsburg: Naturbuch-Verlag. 352 pp.

S19. Schlick-Steiner BC, Steiner FM, Schödl S, Seifert B (2003) *Lasius austriacus* sp.n., a Central European ant related to the invasive *Lasius neglectus*. Sociobiology 41: 725-736.

S20. Seifert B (2003) The ant genus *Cardiocondyla* (Hymenoptera: Formicidae) - a taxonomic revision of the *elegans*, *bulgarica*, *batesii*, *nuda*, *shuckardi*, *stambuloffii*, *wroughtonii*, *emeryi*, and *minutior* species groups. Ann Naturhist Mus Wien 104: 203-338.

S21. Seifert B (2002) How to distinguish most similar insect species - improving the stereomicroscopic and mathematical evaluation of external characters by example of ants. J Appl Entomol 126: 445-454.

S22. Schlick-Steiner BC, Seifert B, Stauffer C, Christian E, Crozier RH, et al. (2007) Without morphology, cryptic species stay in taxonomic crypsis following discovery. Trends Ecol Evol 22: 391-392.

S23. Hölldobler B, Wilson EO (1990) The Ants. Cambridge: Harvard University Press. 732 pp.
