# Supplementary material for: The Evolution of Invasiveness in Garden Ants
Source: PLoS One. 2008 Dec 3;3(12):e3838. doi: 10.1371/journal.pone.0003838 (PMC2585788; doi:10.1371/journal.pone.0003838)
Supplement: Methods S6 — Comparison of Geographical, Genetic and Chemical Distances (0.03 MB DOC) [file pone.0003838.s009.doc]

# Methods S6

## Comparison of Geographical, Genetic and Chemical Distances

In order to compare the relationships between the geographical, genetic and chemical distances between pairs of nests within each group, a modified version of the Mantel test was performed using specially written software (Programmed in RealBASIC 2008; the program is available from http://www.bi.ku.dk/drnash/programs). Since samples were drawn from nests that made up populations, tests in which only the between-population and within-population comparisons were considered were performed in addition to the normal Mantel test of pairwise association. Mantel tests were based on the product-moment correlation coefficient and 10,000 randomizations of the rows and columns of the appropriate (sub)matrices, and probabilities reported are two-tailed. The relationship between each pair of variables was also described in terms of the slope of the least-squares regression line (again calculated separately for within and between population comparisons, as well as over all pairs). Approximate standard errors for this slope were estimated using the delete-a-group jack-knife [S24], based on the deletion of one whole population from the data set at a time. This jack-knifing procedure was found to give less bias in mean estimates than the alternative Tukey jack-knife. The jack-knife procedure was also used to obtain predicted values of the dependent variable (plus its approximate standard error) in each pairwise comparison at a given value of the predictor variable. This was done since the range of the predictor variables was not equal for each of the comparisons made. For example, sampling of *L. neglectus* was carried out over a greater geographic range than sampling of *L. turcicus*. To compare the between-population chemical dissimilarity of the two species, the predicted chemical dissimilarity at the mean geographic separation of between-population *L. neglectus* nest pairs (626 km) was therefore used (Fig. 4B). Likewise, comparisons of the chemical dissimilarity predicted from the genetic distance were based on the mean proportion of unshared alleles in *L. neglectus* nest pairs (0.495; Fig. 4D). Geographical distances were Box-Cox transformed to normalize their distribution and reduce biases in within- versus between-population effects. Welch’s *t*-tests based on the estimated standard errors were then used to assess the differences between *L. neglectus* and the two forms of *L. turcicus.*

For comparing genetic and chemical dissimilarity, the lower allelic richness among *L. neglectus* nests (see main text) could potentially lead to lower than expected genetic dissimilarities and, if surface hydrocarbon profiles are largely genetically determined, lowered chemical dissimilarity, via sampling of a restricted number of alleles rather than through real population-level differences in genetic and chemical diversity. To control for this, we also repeated this analysis only using those nests of *L. turcicus* with allelic richness within the range shown by *L. neglectus* (26 nests from 10 populations for lowland *L. turcicus*, 32 nests from 12 populations for highland *L. turcicus*; between-group comparison of nest-based allelic richness: *F*_2,91_ = 2.16, *P* = 0.122). This produced almost identical relationships to those found using all sampled nests of *L. turcicus* (see Fig. 4D and Table S4).

## References

S24. Kott PS (2001) The delete-a-group jackknife. J Off Stat 17: 521-526.
