# Supplementary material for: The Evolution of Invasiveness in Garden Ants
Source: PLoS One. 2008 Dec 3;3(12):e3838. doi: 10.1371/journal.pone.0003838 (PMC2585788; doi:10.1371/journal.pone.0003838)
Supplement: Methods S7 — Parasite Prevalence (0.03 MB DOC) [file pone.0003838.s010.doc]

# Methods S7

## Parasite Prevalence

*Wolbachia*

Infection with the intracellular bacterium *Wolbachia* in the field was determined for 143 nests from 30 populations (*L. neglectus*: N2, N3, N4, N5, N6, N7, N9, N11, N13, and N14; lowland *L. turcicus*: T2, T3, T5, T6, T7, T8, T9, T10, T11, and T12; highland *L. turcicus*: T13, T14, T16, T19, T21, T22, T23, T24, and T25; 4.8 ± 0.5 s.d. nests per population, 1 worker per nest). We performed conventional PCR amplification using the *wsp* gene of *Wolbachia* and scored infection as either present or absent [S25]. For all samples, for which conventional PCR showed absence of infection, we used real time fluorescence detection quantitative PCR because of its demonstrated superior sensitivity to low template concentration, using a Roche 2.0 LightCycler machine and SYBR green (Roche Biochemicals [S26]). This revealed that quantitative PCR was 37% more sensitive than conventional PCR. In the latter case we used the *Wolbachia* single copy gene *ftsZ* since it is more conserved and allowed accurate identification of PCR products through melting point analysis. Melting curves were used to confirm the presence or absence of *Wolbachia* products. We sequenced a sample of 15 products to verify amplification specificity.

*Beauveria*

Dead worker ants were collected during the first week after the ants had been brought into the laboratory (881 individuals from 27 populations: *L. neglectus*: N2, N3, N4, N6, N7, N11, N12, N13, N14, and N18; lowland *L. turcicus*: T1, T2, T3, T4, T5, T6, T7, T8, T9, and T10; highland *L. turcicus*: T13, T14, T18, T19, T21, T23, and T24; 32.6 ± 16.1 s.d. individuals from at least 3 nests per population). These were transferred to Petri dishes with damp filter paper and kept at 23°C with frequent watering to keep humidity high. After three weeks the presence or absence of fungal spores on the body surface was determined for each individual. The prevalence of the entomopathogenic fungus *Beauveria bassiana* was determined. Differences in population prevalence between species were tested with a logistic regression model with binomial error structure applying the GENMOD procedure in SAS/enterprise while estimating the scaling parameter by the square root of DEVIANCE/DOF.

## References

S25. Reuter M, Pedersen JS, Keller L (2005) Loss of *Wolbachia* infection during colonisation in the invasive Argentine ant *Linepithema humile*. Heredity 94: 364-369.

S26. Fenollar F, Maurin M, Raoult D (2003) *Wolbachia pipientis* growth kinetics and susceptibilities to 13 antibiotics determined by immunofluorescence staining and Real-Time PCR. Antimicrob Agents Chemother 47: 1665-1671.
