# Supplementary material for: The Evolution of Invasiveness in Garden Ants
Source: PLoS One. 2008 Dec 3;3(12):e3838. doi: 10.1371/journal.pone.0003838 (PMC2585788; doi:10.1371/journal.pone.0003838)
Supplement: Table S1 — Population localities: countries, names, coordinates and altitudes of the populations. (0.14 MB DOC) [file pone.0003838.s011.doc]

# Table S1

| **Species** | **form** | **ID** | **Country** | **Locality** | **Latitude** | **Longitude** | **Altitude [m]** |
| --- | --- | --- | --- | --- | --- | --- | --- |
| ***L. neglectus*** |  | N1 | Turkey | İstanbul | 41°00' N | 28°59' E | 25 |
|  |  | N2 | Germany | Jena | 50°55' N | 11°35' E | 151 |
|  |  | N3 | Spain | Seva | 41°50' N | 2°16' E | 750 |
|  |  | N4 | France | Toulouse | 43°36' N | 1°26' E | 151 |
|  |  | N5 | France | Paris | 48°42' N | 2°08' E | 35 |
|  |  | N6 | Belgium | Ghent | 51°02' N | 3°44' E | 4 |
|  |  | N7 | Turkey | Edirne 2 | 41°40' N | 26°33' E | 72 |
|  |  | N8 | Hungary | Budapest | 47°29' N | 19°02' E | 91 |
|  |  | N9 | Italy | Volterra | 43°24' N | 10°51' E | 575 |
|  |  | N10 | Hungary | Debrecen | 47°32' N | 21°37' E | 121 |
|  |  | N11 | Turkey | Edirne 1 | 43°26' N | 26°34' E | 47 |
|  |  | N12 | Turkey | Edirne 3 | 41°39' N | 26°35' E | 51 |
|  |  | N13 | Spain | Bellaterra | 41°29' N | 2°06' E | 90 |
|  |  | N14 | Turkey | Bayramiç | 39°48' N | 26°36' E | 101 |
|  |  | N15 | Poland | Warsaw | 52°14' N | 21°01' E | 98 |
|  |  | N16 | Kyrgyzstan | Bishkek | 42°87' N | 74°60' E | 771 |
|  |  | N17 | Romania | Baile Herculane-Dobreta | 44°37' N | 28°20' E | 201 |
|  |  | N18 | Bulgaria | Kavarna | 52°14' N | 21°01' E | 120 |
|  |  |  |  |  |  |  |  |
| ***L. turcicus*** | low | T1 | Turkey | Müezzinler | 40°58' N | 30°19' E | 68 |
|  | low | T2 | Turkey | Ağva | 41°08' N | 29°51' E | 1 |
|  | low | T3 | Turkey | Bayramiç | 39°48' N | 26°36' E | 101 |
|  | low | T4 | Turkey | Pınarhisar | 41°37' N | 27°31' E | 237 |
|  | low | T5 | Turkey | Yalova | 40°13' N | 26°25' E | 8 |
|  | low | T6 | Turkey | Kırklareli | 41°44' N | 27°13' E | 210 |
|  | low | T7 | Turkey | Hacıaslanlar Çayı | 39°40' N | 27°06' E | 370 |
|  | low | T8 | Turkey | Yassıbağ | 39°45' N | 26°39' E | 328 |
|  | low | T9 | Turkey | Külcüler | 39°48' N | 26°46' E | 237 |
|  | low | T10 | Turkey | Evciler | 39°47' N | 26°41' E | 161 |
|  | low | T11 | Turkey | Ödemiş | 38°13' N | 27°58' E | 118 |
|  | low | T12 | Turkey | Beydağ | 38°09' N | 28°07' E | 156 |
|  | high | T13 | Turkey | Yeşilhisar | 38°38' N | 29°54' E | 1116 |
|  | high | T14 | Turkey | Pazaryeri | 39°55' N | 29°59' E | 724 |
|  | high | T15 | Turkey | Karaçam | 40°38' N | 30°20' E | 60 |
|  | high | T16 | Turkey | Hayrettin | 40°29' N | 30°05' E | 94 |
|  | high | T17 | Turkey | Bilecik | 40°08' N | 29°58' E | 509 |
|  | high | T18 | Turkey | Uşak | 38°40' N | 29°24' E | 906 |
|  | high | T19 | Turkey | Kütahya | 39°25' N | 29°59' E | 940 |
|  | high | T20 | Turkey | Inönü | 39°44' N | 30°10' E | 1007 |
|  | high | T21 | Turkey | Afyon | 38°45' N | 30°32' E | 1026 |
|  | high | T22 | Turkey | Akşehir | 38°21' N | 31°24' E | 1021 |
|  | high | T23 | Turkey | Maltepe | 38°36' N | 30°52' E | 994 |
|  | high | T24 | Turkey | Bağkonak | 38°13' N | 31°16' E | 1170 |
|  | high | T25 | Turkey | Isparta | 37°39' N | 30°43' E | 715 |
