# Supplementary material for: The Evolution of Invasiveness in Garden Ants
Source: PLoS One. 2008 Dec 3;3(12):e3838. doi: 10.1371/journal.pone.0003838 (PMC2585788; doi:10.1371/journal.pone.0003838)
Supplement: Table S2 — List of cuticular hydrocarbon compounds and their mean proportional peak areas in the three profile types (coded as white, grey or black squares in Fig. 2A) (0.09 MB DOC) [file pone.0003838.s012.doc]

# Table S2

| **Peak no.** | **Compound abbreviation** | **Full compound name** | ***Ln* 17 populations** | | **Lowland *Lt* 12 populations** | | **Highland *Lt* 13 populations** | |
| --- | --- | --- | --- | --- | --- | --- | --- | --- |
|  |  |  | **Mean** | **s.d.** | **Mean** | **s.d.** | **Mean** | **s.d.** |
| 1 | C31:2 | hentriacontadiene | - | - | 0.2 | 0.3 | 0.3 | 0.4 |
| 2 | 11C31:1 | hentriacont-11-ene | 0.4 | 0.2 | 1.2 | 1.2 | 5.7 | 6.0 |
| 3 | C31 | hentriacontane | 0.6 | 0.4 | 1.7 | 1.3 | 2.2 | 1.2 |
| 4 | 13MeC31 | 13Methylhentriacontane | 0.4 | 0.2 | 0.7 | 1.0 | 2.1 | 1.8 |
| 5 | 3MeC31 | 3Methylhentriacontane | 1.5 | 0.5 | 0.4 | 0.4 | 0.8 | 0.6 |
| 6 | C33:2 | tritriacontadiene | - | - | 0.2 | 0.3 | 0.0 | 0.1 |
| 7 | C33:2 | tritriacontadiene | 7.6 | 1.6 | 3.0 | 1.2 | 6.8 | 3.9 |
| 8 | C33:2 | tritriacontadiene | 2.3 | 0.8 | 16.3 | 2.7 | 7.9 | 3.6 |
| 9 | C33:2 | tritriacontadiene | 2.2 | 1.3 | 3.4 | 3.1 | 2.1 | 0.9 |
| 10 | 12,13C33:1 | tritriacont-12-ene +  tritriacont-13-ene | 10.7 | 2.1 | 5.9 | 6.4 | 1.2 | 2.4 |
| 11 | 10,11C33:1 | tritriacont-10-ene +  tritriacont-11-ene | 7.6 | 2.1 | 8.4 | 3.2 | 12.0 | 4.5 |
| 12 | 7C33:1 | tritriacont-7-ene | 1.2 | 0.4 | 2.8 | 1.1 | 1.7 | 1.2 |
| 13 | 13Me21,23C33:1 | 13Methyltritriacont-21-ene + 13Methyltritriacont-23-ene | 11.4 | 3.5 | 18.4 | 7.0 | 40.5 | 6.0 |
| 14 | 13,15MeC33 | 13Methyltritriacontane + 15Methyltritriacontane | 4.9 | 1.4 | 1.1 | 0.6 | 2.9 | 0.8 |
| 15 | 3Me21C33:1 | 3Methyltritriacont-21-ene | 2.4 | 0.8 | 0.2 | 0.2 | 0.5 | 0.5 |
| 16 | 3Me23C33:1 | 3Methyltritriacont-23-ene | 2.3 | 1.0 | 0.4 | 0.2 | 0.6 | 0.3 |
| 17 | 11,23diMeC33 | 11,23Dimethyltritriacontane | 0.1 | 0.3 | - | - | - | - |
| 18 | 3MeC33 + 5,15diMeC33 | 3Methyltritriacontane + 5,15Dimethyltritriacontane | 1.7 | 0.8 | 0.1 | 0.1 | 0.1 | 0.2 |
| 19 | 12,14,22Me21,23C34:1 | 12,14,22Methyltetratriacont-21-ene + 12,14,22Methyltetratriacont-23-ene | 1.2 | 0.3 | 1.1 | 0.7 | 1.1 | 0.7 |
| 20 | C35:2 | pentatriacontadiene | 10.7 | 2.2 | 0.4 | 0.5 | 0.2 | 0.1 |
| 21 | C35:2 | pentatriacontadiene | 1.7 | 0.6 | 19.1 | 7.5 | 4.8 | 5.6 |
| 22 | C35:2 | pentatriacontadiene | 0.9 | 0.3 | 6.0 | 4.1 | 0.9 | 0.9 |
| 23 | 21C35:1 | pentatriacont-21-ene | 1.0 | 0.5 | 1.8 | 0.6 | 0.4 | 0.5 |
| 24 | 23C35:1 | pentatriacont-23-ene | 1.2 | 0.7 | 1.3 | 0.4 | 0.4 | 0.4 |
| 25 | 13,15Me21,23C35:1 | 13,15Methylpentatriacont-21-ene + 13,15Methylpentatriacont-23-ene | 25.4 | 4.0 | 5.9 | 2.8 | 4.6 | 2.6 |
| 26 | 13MeC35 + 15MeC35 | 13Methylpentatriacontane + 15Methylpentatriacontane | 0.7 | 0.3 | 0.1 | 0.3 | - | - |
