# Supplementary material for: The Evolution of Invasiveness in Garden Ants
Source: PLoS One. 2008 Dec 3;3(12):e3838. doi: 10.1371/journal.pone.0003838 (PMC2585788; doi:10.1371/journal.pone.0003838)
Supplement: Table S3 — Morphological parameters measured for workers of L. neglectus, lowland and highland L. turcicus (mean±s.d. of measurements of the three groups, derived from population means, n = 18, 12, and 13 populations). (0.06 MB DOC) [file pone.0003838.s013.doc]

# Table S3

| **Morphological parameter** | ***L. neglectus*** | | **Lowland *L. turcicus*** | | **Highland *L. turcicus*** | |
| --- | --- | --- | --- | --- | --- | --- |
|  | **Mean** | **s.d.** | **Mean** | **s.d.** | **Mean** | **s.d.** |
| Head size | 0.772 | 0.027 | 0.778 | 0.037 | 0.837 | 0.030 |
| Cephalic length/width* | 1.092 | 0.007 | 1.079 | 0.010 | 1.085 | 0.007 |
| Scape length* | 0.964 | 0.011 | 0.948 | 0.011 | 0.966 | 0.014 |
| Distance clypeus-antennal socket* | 4.133 | 0.255 | 3.550 | 0.234 | 3.986 | 0.160 |
| Eye size index* | 0.242 | 0.003 | 0.235 | 0.004 | 0.237 | 0.004 |
| Postocular distance* | 0.226 | 0.004 | 0.237 | 0.004 | 0.226 | 0.004 |
| Length of pronotal setae* | 0.123 | 0.005 | 0.125 | 0.005 | 0.125 | 0.004 |
| Length of gulal setae* | 0.111 | 0.007 | 0.120 | 0.004 | 0.125 | 0.006 |
| Number of gulal setae* | 2.793 | 0.551 | 3.277 | 0.361 | 3.992 | 0.350 |
| Number of hind tibia setae* | 0.239 | 0.253 | 0.159 | 0.108 | 0.352 | 0.304 |
| Number of occipital setae* | 8.994 | 1.253 | 5.367 | 0.574 | 7.412 | 1.710 |
| Number of scape setae* | 0.064 | 0.095 | 0.034 | 0.054 | 0.034 | 0.049 |
| Number of subspiracular setae* | 3.286 | 0.602 | 3.656 | 0.487 | 4.108 | 0.708 |
| Clypeal pubescence distance* | 5.459 | 0.328 | 5.142 | 0.142 | 5.337 | 0.337 |

Measurements corrected for allometry are indicated by *. All size measurements are in mm.
