# Supplementary material for: The Evolution of Invasiveness in Garden Ants
Source: PLoS One. 2008 Dec 3;3(12):e3838. doi: 10.1371/journal.pone.0003838 (PMC2585788; doi:10.1371/journal.pone.0003838)
Supplement: Table S4 — Summary of regression analyses of chemical dissimilarity as a function of geographical distance and genetic dissimilarity. (0.10 MB DOC) [file pone.0003838.s014.doc]

# Table S4

| **Relationship between geographic distance (box-cox transformed) and chemical dissimilarity** | | | | |
| --- | --- | --- | --- | --- |
|  |  |  |  |  |
|  |  | Across all pairs | Within-population pairs | Between-population pairs |
| ***Lasius neglectus*** | | | | |
|  | Mantel *P* | <0.0001 | <0.0001 | 0.0731 |
|  | Slope ± SE | 0.0022 ± 0.0012 | 0.0283 ± 0.0246 | 0.0015 ± 0.0019 |
|  | *Predicted Y* ± SE |  |  | 2.896 ± 0.7587 |
|  |  |  |  |  |
| **Lowland *Lasius turcicus*** | | | | |
|  | Mantel *P* | <0.0001 | <0.0001 | 0.0025 |
|  | Slope ± SE | 0.0106 ± 0.0051 | 0.0332 ± 0.0193 | 0.0120 ± 0.0117 |
|  | *Predicted Y* ± SE |  |  | 10.67 ± 2.47 |
|  |  |  |  |  |
| **Highland *Lasius turcicus*** | | | | |
|  | Mantel *P* | <0.0001 | <0.0001 | <0.0001 |
|  | Slope ± SE | 0.0091 ± 0.0033 | 0.0306 ± 0.0314 | 0.0196 ± 0.0100 |
|  | *Predicted Y* ± SE |  |  | 11.13 ± 2.02 |
|  |  |  |  |  |
|  |  |  |  |  |
| **Relationship between genetic dissimilarity and chemical dissimilarity** | | | | |
|  |  |  |  |  |
|  |  | Across all pairs | Within-population pairs | Between-population pairs |
| ***Lasius neglectus*** | | | | |
|  | Mantel *P* | <0.0001 | 0.7497 | 0.0157 |
|  | Slope ± SE | 3.024 ± 0.992 | 0.2200 ± 2.683 | 2.234 ± 1.946 |
|  | *Predicted Y* ± SE | 1.918 ± 0.658 |  |  |
|  |  |  |  |  |
| **Lowland *Lasius turcicus*** | | | | |
|  | Mantel *P* | <0.0001 | <0.0001 | <0.0001 |
|  | Slope ± SE | 13.13 ± 4.06 | 9.53 ± 3.03 | 13.71 ± 6.02 |
|  | *Predicted Y* ± SE | 7.681 ± 0.936 |  |  |
|  |  |  |  |  |
| **Highland *Lasius turcicus*** | | | | |
|  | Mantel *P* | <0.0001 | <0.0001 | 0.0007 |
|  | Slope ± SE | 6.594 ± 2.743 | 7.250 ± 2.890 | 5.475 ± 2.826 |
|  | *Predicted Y* ± SE | 6.459 ± 0.407 |  |  |
|  |  |  |  |  |
| **Lowland *Lasius turcicus* (restricted allelic richness)** | | | | |
|  | Mantel *P* | <0.0001 | <0.0001 | <0.0001 |
|  | Slope ± SE | 12.77 ± 3.71 | 10.17 ± 3.77 | 12.44 ± 4.78 |
|  | *Predicted Y* ± SE | 8.361 ± 0.866 |  |  |
|  |  |  |  |  |
| **Highland *Lasius turcicus* (restricted allelic richness)** | | |  |  |
|  | Mantel *P* | 0.0005 | <0.0001 | 0.0067 |
|  | Slope ± SE | 6.934 ± 3.696 | 8.538 ± 5.997 | 5.824 ± 4.121 |
|  | *Predicted Y* ± SE | 6.268 ± 0.776 |  |  |

Significance of the relationship was assessed by Mantel tests, and means and standard errors were estimated by jack-knifing over populations. *Predicted Y* is the chemical dissimilarity predicted at the mean value of the predictor variable for *L. neglectus* (mean geographic distance = 626 km; mean genetic dissimilarity = 0.495).
